# Supplementary material for: TIAToolbox as an end-to-end library for advanced tissue image analytics
Source: Commun Med (Lond). 2022 Sep 24;2:120. doi: 10.1038/s43856-022-00186-5 (PMC9509319; doi:10.1038/s43856-022-00186-5)
Supplement: Supplementary file 1 — Supplementary Material [file 43856_2022_186_MOESM1_ESM.pdf]

Supplementary Material

Supplementary Note 1

```
# Implementation without TIAToolbox (16 lines)
import numpy as np
import openslide

PATCH_SIZE = (224, 224)

slide = openslide.OpenSlide("CMU-1-Small-Region.svs")
mpp = np.array([
  float(slide.properties["openslide.mpp-x"]),
  float(slide.properties["openslide.mpp-y"]),
])
target_mpp = np.ones(2)
scale_factor = target_mpp / mpp
read_patch_size = np.multiply(PATCH_SIZE, scale_factor).astype(int)
mosaic_shape = np.divide(slide.dimensions, read_patch_size).astype(int)

for ij in np.ndindex(tuple(mosaic_shape)):
  xy = (np.multiply(ij, PATCH_SIZE) * scale_factor).astype(int)
  patch = slide.read_region(tuple(xy), 0, tuple(read_patch_size)).convert("RGB")
  patch = patch.resize(PATCH_SIZE)
  if np.percentile(np.mean(patch, axis=-1), 5) > 200:
    continue
  patch.save(f"patches/{'-'.join(str(c) for c in xy)}.jpeg")

# TIAToolbox based implementation (5 lines)
from tiatoolbox.tools.patchextraction import SlidingWindowPatchExtractor
from matplotlib import pyplot as plt

extractor = SlidingWindowPatchExtractor(
  "CMU-1-Small-Region.svs",
  (224, 224),
  resolution=1,
  units="mpp",
)
for n, patch in enumerate(extractor):
    plt.imsave(f"patches/{n}.jpeg", patch)
```

Source code demonstrating how the toolbox can simplify implementation of a common task such as patch extraction. The implementation without using TIAToolbox has 16 lines of code, and the implementation with TIAToolbox has only 5 lines of code. Additionally, the implementation without the toolbox does not work with formats which are not supported by OpenSlide, whereas the version using the TIAToolbox extractor class supports many more WSI file types.

Supplementary Note 2

```
037 from tiatoolbox.wsicore.wsireader import WSIReader
038
039 tissue = WSIReader.open("path/wsi1.svs")
040 mask = tissue.tissue_mask()
041
042 tissue_region, mask_region = (
043     wsi.read_rect(
044         location=(0, 0),
045         size=(512, 512),
046         resolution=0.5,
047         units="mpp",
048     )
049     for wsi in (tissue, mask)
050 )
```

A short Python expression demonstrating a synchronous reading of a tissue WSI and a lower resolution mask derived from the tissue WSI. Both read operations use the same function call arguments coordinates, including identical coordinates, as shown on lines 008 through 011. This is despite the underlying image data being stored at different resolutions. This demonstrates the power of the VirtualWSIReader object to enable easy reading from both a WSI and a derived image, such as a tissue mask, which may be internally represented at a different resolution.

Supplementary Note 3

```
051 from tiatoolbox.models.engine.patch_predictor import PatchPredictor
052
053 data = [img1, img2]  # input arrays as a list
054 predictor = PatchPredictor(
055   pretrained model="resnet18-kather100k",
056 )
057 output = predictor.predict(
058   data,
059   mode="patch",
060 )
```

An example Python script for patch prediction using TIAToolbox using pre-extracted patches as input.

Supplementary Note 4

```
061 from tiatoolbox.models.engine.patch_predictor import PatchPredictor
062
063 # Input WSI file paths
064 data = ["path/wsi1.svs", "path/wsi2.svs"]
065
066 predictor = PatchPredictor(
067   pretrained_model="resnet18-kather100k"
068 )
069
070 output = predictor.predict(
071   data,
072   mode="wsi",
073 )
```

An example Python script for patch prediction using TIAToolbox using whole-slide images as input. The same API is used for image tiles by changing the mode to “tile” in the predict method.

Supplementary Note 5

```
from tiatoolbox.models.engine.semantic_segmentor import SemanticSegmentor

# Input WSI file paths
data = ["path/wsi1.svs", "path/wsi2.svs"]

segmentor = SemanticSegmentor(
  pretrained_model="fcn_resnet50_unet-bcss",
)

# WSI prediction
output = segmentor.predict(
  imgs=[wsi_file_name],
  mode="wsi",
)
```

Supplying a WSI as input to a semantic segmentation model. Here, we use a U-Net with a ResNet50 backbone that is trained on the breast cancer semantic segmentation (BCSS) dataset.

Supplementary Note 6

```
from tiatoolbox.models.engine.nucleus_instance_segmentor import NucleusInstanceSegmentor

# Input WSI file paths
data = ["path/wsi1.svs", "path/wsi2.svs"]

# Instantiate the nucleus instance segmentor
inst_segmentor = NucleusInstanceSegmentor(
  pretrained_model="hovernet_fast-pannuke",
)

# WSI prediction
wsi_output = inst_segmentor.predict(
  [wsi_file_name],
  mode="wsi",
)
```

Supplying a WSI to a nucleus instance segmentation model. Here, we use a HoVer-Net trained on the PanNuke dataset.

Supplementary Note 7

```
from tiatoolbox.models.engine.patch_predictor import PatchPredictor

predictor = PatchPredictor(
  pretrained model="resnet18-kather100k",
  pretrained_weights="path/weights.pth",
)
```

Code showing Python script of how to use your own pretrained weights, rather than those provided by TIAToolbox.

Supplementary Note 8

```
import numpy as np
from matplotlib import pyplot as plt

from tiatoolbox.tools.graph import SlideGraphConstructor

# Load XY patch positions in the WSI
positions = np.load(f"path/wsi1.position.npy")
# Load features for each patch
features = np.load(f"path/wsi1.features.npy")

constructor = SlideGraphConstructor()
graph = constructor.build(
  positions[:, :2],
  features,
  feature_range_thresh=None,
)

constructor.visualise(graph)
plt.show()
```

Building and visualizing a slide graph from a set of patch locations and associated features.

Supplementary Note 9

```
from tiatoolbox.wsiscore.wsireader import WSIReader
from tiatoolbox.visualization.tileserver import TileServer

wsi = WSIReader.open("path/wsi1.svs")
app = TileServer(
  title="Testing TileServer",
  layers={
    "wsi1": wsi,
  },
)
app.run()
```

Creating a tile server web server gateway interface (WSGI) application and running a simple development HTTP server to display two WSI images in a web browser.

Supplementary Note 10

```python
from shapely.geometry import Polygon
from tiatoolbox.annotation.storage import SQLiteStore, Annotation

# Store a simple triangle annotation
store = SQLiteStore("annotations.db")
annotation = Annotation(
  geometry=Polygon([(0, 0), (1, 0), (0, 1)]),
  properties={},
)
uuid = store.append(annotation)

# Access the annotation and add some properties
print(store[uuid])
annotation.properties = {"class": 1, "foo": "bar"}
store[uuid] = Annotation(triangle)

# Change just part of the properties
store.patch(uuid, {"class": 2})

# Query in a bounding box (left, right, top, bottom)
uuids = store.iquery([0, 0, 1, 1])

# Query with a predicate statement
results = store.query([0, 0, 1, 1], where="props['class']==2")
```

An example of creating an annotation and storing it in an SQLite database. The annotation is given a universally unique identifier (UUID) as no key was specified. This is used to look up the annotation and modify it. Also shown here, is how to query for annotations using a bounding box and adding a predicate statement to filter the results.

Supplementary Note 11

```python
import numpy as np

from tiatoolbox.models.engine.patch_predictor import PatchPredictor
from tiatoolbox.utils.misc import imwrite

WSI_PATH = "path/wsi1.svs"

# Tumour detection
tumour_predictor = PatchPredictor(
  pretrained_model='resnet18-idars-tumour',
)

tumour_output = tumour_predictor.predict(
  imgs=[WSI_PATH],
  mode='wsi',
)

tumour_mask = tumour_predictor.merge_predictions(
  WSI_PATH,
  tumour_output[0],
  resolution=5,
  units="power",
)
tumour_mask = tumour_mask == 2  # Binarise the output
imwrite('tumour_mask.png', tumour_mask.astype('uint8'))

# WSI Prediction
msi_predictor = PatchPredictor(
  pretrained_model='resnet34-idars-msi',
)

msi_output = msi_predictor.predict(
  imgs=[WSI_PATH],
  masks=['tumour_mask.png'],
  mode='wsi',
  return_probabilities=True,
)

# Slide-Level Score:
# Only consider MSI class
msi_probabilities = np.array(msi_output[0]['probabilities'])[...,1]
# Get the average over all tumour tiles
average_msi_probability = np.mean(msi_probabilities)
```

IDaRS inference using TIAToolbox. Here, we demonstrate that we can simplify the overall inference pipeline without the need for many lines of code. The following steps are performed: 1) tumor detection, 2) saving tumor mask, 3) mutation prediction in tumor regions and 4) patch aggregation.

Supplementary Table 1

Detailed metrics of Models for patch prediction.

Model Family	Architecture Variants	Training Dataset(s)	Metric(s)
**AlexNet[1]**	AlexNet	Kather 100k[2], PCam[3]	Supplementary Table 2
**ResNet[4]**	ResNet-18, ResNet-34, ResNet-50, ResNet-101	Kather 100k[2], PCam[3]	Supplementary Table 2
**ResNeXt[5]**	ResNeXt-50, ResNeXt-101	Kather 100k[2], PCam[3]	Supplementary Table 2
**Wide ResNet[6]**	Wide ResNet-50, Wide ResNet101	Kather 100k[2], PCam[3]	Supplementary Table 2
**DenseNet[7]**	DenseNet121, DenseNet161, DenseNet169, DenseNet201	Kather 100k[2], PCam[3]	Supplementary Table 2
**MobileNet[8,9]**	MobileNet-v2, MobileNet-v3 small, MobileNet-v3 large	Kather 100k[2], PCam[3]	Supplementary Table 2
**GoogLeNet[10]**	GoogLeNet	Kather 100k[2], PCam[3]	Supplementary Table 2

Supplementary Table 2

Patch classification performance of models provided by TIAToolbox on both the Kather100K and Patch Camelyon (PCam) datasets.

	Kather100K F_1	PCam F_1
**AlexNet[1]**	0.965	0.840
**ResNet18[4]**	0.990	0.888
**ResNet34[4]**	0.991	0.889
**ResNet50[4]**	0.989	0.892
**ResNet101[4]**	0.989	0.888
**ResNeXt50 32x4d[5]**	0.992	0.900
**ResNeXt101 32x8d[5]**	0.991	0.892
**Wide ResNet50[6]**	0.989	0.901
**Wide ResNet101[6]**	0.990	0.898
**DenseNet121[7]**	0.993	0.897
**DenseNet161[7]**	0.992	0.893
**DenseNet169[7]**	0.992	0.895
**DenseNet201[7]**	0.991	0.891
**GoogLeNet[10]**	0.990	0.899
**MobileNet v2[8,9]**	0.991	0.895
**MobileNet v3 large[8,9]**	0.992	0.890
**MobileNet v3 small[8,9]**	0.992	0.867

Supplementary Table 3

Semantic segmentation performance (Sørensen–Dice score) on the Breast Cancer Semantic Segmentation (BCSS) dataset. Here, we use a U-Net model with a ResNet50 encoder.

	Tumor	Stroma	Inflammatory	Necrosis	Other	All
**Amgad *et al.*[11]**	0.851	0.800	0.712	0.723	0.666	0.750
**TIAToolbox**	0.885	0.825	0.761	0.765	0.581	0.763

Supplementary Table 4

Performance of IDaRS provided as part of TIAToolbox, compared to the original implementation.

	MSI	TP53	BRAF	CIMP	CIN	HM
**Bilal *et al.*[12]**	0.828	0.755	0.813	0.853	0.860	0.846
**TIAToolbox**	0.870	0.747	0.750	0.748	0.810	0.790

Supplementary Table 5

Performance of SlideGraph+ using five-fold cross-validation provided as part of TIAToolbox, compared to the original implementation. Here we report the mean and the standard deviation across the folds.

	HER2	ER
**Lu *et al.* (original)**	0.710±0.020	-
**TIAToolbox**	0.738±0.043	0.872±0.023

Supplementary Table 6

Detailed metrics of Models for semantic segmentation.

Model Family	Architecture Variants	Training Dataset(s)	Metric(s)
**UNet[13]**	ResNet-50 backbone	TCGA-BCSS	Supplementary Table 3
**HoVer-Net[14]**	HoVer-Net+[15]	Private oral dysplasia cohort (not available)	Shephard *et al.*[15]

Supplementary Table 7

Detailed metrics of Models for nucleus segmentation or classification.

Model Family	Architecture Variants	Training Dataset(s)	Metric(s)
**HoVer-Net[14]**	Original,<br>Fast,<br>HoVer-Net+	Kumar (MoNuSeg Subset)[16],<br>PanNuke[17,18],<br>CoNSeP[14],<br>MoNuSAC[19],<br>Private oral dysplasia cohort (not available)	Graham *et al.*[14],<br>Gamper *et al.*[18],<br>Shephard *et al.*[15],

Supplementary Table 8

Whole slide classification.

Model Family	Architecture Variants	Training Dataset(s)	Metric(s)
**IDaRS**	ResNet-18 (tumor)<br>ResNet-34 (mutation)	TCGA-COAD	Supplementary Table 4
**SlideGraph**	SlideGraph+	TCGA-BRCA	Supplementary Table 5,<br>Lu *et al.*[20]

Supplementary References

1. Krizhevsky, A., Sutskever, I. & Hinton, G.E. Imagenet classification with deep convolutional neural networks. *Advances in neural information processing systems* **25**, 1097-1105 (2012).
2. Kather, J.N.*, et al.* Predicting survival from colorectal cancer histology slides using deep learning: A retrospective multicenter study. *PLoS medicine* **16**, e1002730 (2019).
3. Veeling, B.S., Linmans, J., Winkens, J., Cohen, T. & Welling, M. Rotation equivariant cnns for digital pathology. in *International Conference on Medical image computing and computer-assisted intervention* 210-218 (Springer, 2018).
4. He, K., Zhang, X., Ren, S. & Sun, J. Deep residual learning for image recognition. in *Proceedings of the IEEE conference on computer vision and pattern recognition* 770-778 (2016).
5. Xie, S., Girshick, R., Dollár, P., Tu, Z. & He, K. Aggregated residual transformations for deep neural networks. in *Proceedings of the IEEE conference on computer vision and pattern recognition* 1492-1500 (2017).
6. Zagoruyko, S. & Komodakis, N. Wide residual networks. *Preprint at* *https://arxiv.org/abs/1605.07146* (2016).
7. Huang, G., Liu, Z., Van Der Maaten, L. & Weinberger, K.Q. Densely connected convolutional networks. in *Proceedings of the IEEE conference on computer vision and pattern recognition* 4700-4708 (2017).
8. Howard, A.*, et al.* Searching for mobilenetv3. in *Proceedings of the IEEE/CVF International Conference on Computer Vision* 1314-1324 (2019).
9. Sandler, M., Howard, A., Zhu, M., Zhmoginov, A. & Chen, L.-C. Mobilenetv2: Inverted residuals and linear bottlenecks. in *Proceedings of the IEEE conference on computer vision and pattern recognition* 4510-4520 (2018).
10. Szegedy, C.*, et al.* Going deeper with convolutions. in *Proceedings of the IEEE conference on computer vision and pattern recognition* 1-9 (2015).
11. Amgad, M.*, et al.* Structured crowdsourcing enables convolutional segmentation of histology images. *Bioinformatics* **35**, 3461-3467 (2019).
12. Bilal, M.*, et al.* Development and validation of a weakly supervised deep learning framework to predict the status of molecular pathways and key mutations in colorectal cancer from routine histology images: a retrospective study. *The Lancet Digital Health* **3**(2021).
13. Long, J., Shelhamer, E. & Darrell, T. Fully convolutional networks for semantic segmentation. in *Proceedings of the IEEE conference on computer vision and pattern recognition* 3431-3440 (2015).
14. Graham, S.*, et al.* Hover-net: Simultaneous segmentation and classification of nuclei in multi-tissue histology images. *Medical Image Analysis* **58**, 101563 (2019).
15. Shephard, A.J.*, et al.* Simultaneous Nuclear Instance and Layer Segmentation in Oral Epithelial Dysplasia. in *Proceedings of the IEEE/CVF International Conference on Computer Vision* 552-561 (2021).
16. Kumar, N.*, et al.* A Multi-Organ Nucleus Segmentation Challenge. *IEEE Transactions on Medical Imaging* **39**, 1380-1391 (2020).
17. Gamper, J., Alemi Koohbanani, N., Benet, K., Khuram, A. & Rajpoot, N. PanNuke: An Open Pan-Cancer Histology Dataset for Nuclei Instance Segmentation and Classification. 11-19 (Springer International Publishing, Cham, 2019).
18. Gamper, J.*, et al.* Pannuke dataset extension, insights and baselines. *Preprint at* *https://arxiv.org/abs/2003.10778* (2020).
19. Verma, R.*, et al.* MoNuSAC2020: A Multi-organ Nuclei Segmentation and Classification Challenge. *IEEE Transactions on Medical Imaging* **40**, 3413 - 3423 (2021).

20. Lu, W., Toss, M., Rakha, E., Rajpoot, N. & Minhas, F. SlideGraph+: Whole Slide Image Level Graphs to Predict HER2Status in Breast Cancer. *Preprint at https://arxiv.org/abs/2110.06042* (2021).
