# Supplementary material for: TIAToolbox as an end-to-end library for advanced tissue image analytics
Source: Commun Med (Lond). 2022 Sep 24;2:120. doi: 10.1038/s43856-022-00186-5 (PMC9509319; doi:10.1038/s43856-022-00186-5)
Supplement: Supplementary file 3 — Reporting Summary [file 43856_2022_186_MOESM3_ESM.pdf]

nature research

Corresponding author(s): Shan E Ahmed Raza
Last updated by author(s): Aug 16, 2022

Reporting Summary

Nature Research wishes to improve the reproducibility of the work that we publish. This form provides structure for consistency and transparency in reporting. For further information on Nature Research policies, see our Editorial Policies and the Editorial Policy Checklist.

Statistics

For all statistical analyses, confirm that the following items are present in the figure legend, table legend, main text, or Methods section.

n/a	Confirmed	
☒	☐	The exact sample size (n) for each experimental group/condition, given as a discrete number and unit of measurement
☒	☐	A statement on whether measurements were taken from distinct samples or whether the same sample was measured repeatedly
☒	☐	The statistical test(s) used AND whether they are one- or two-sided<br>*Only common tests should be described solely by name; describe more complex techniques in the Methods section.*
☒	☐	A description of all covariates tested
☐	☒	A description of any assumptions or corrections, such as tests of normality and adjustment for multiple comparisons
☐	☒	A full description of the statistical parameters including central tendency (e.g. means) or other basic estimates (e.g. regression coefficient) AND variation (e.g. standard deviation) or associated estimates of uncertainty (e.g. confidence intervals)
☒	☐	For null hypothesis testing, the test statistic (e.g. F, t, r) with confidence intervals, effect sizes, degrees of freedom and P value noted<br>*Give P values as exact values whenever suitable.*
☒	☐	For Bayesian analysis, information on the choice of priors and Markov chain Monte Carlo settings
☒	☐	For hierarchical and complex designs, identification of the appropriate level for tests and full reporting of outcomes
☒	☐	Estimates of effect sizes (e.g. Cohen's d, Pearson's r), indicating how they were calculated

Our web collection on statistics for biologists contains articles on many of the points above.

Software and code

Policy information about availability of computer code

Data collection	All the codes and data is publicly available and can be downloaded via a web browser.
Data analysis	The TIAToolbox software library presented in the paper was used for analysis.

For manuscripts utilizing custom algorithms or software that are central to the research but not yet described in published literature, software must be made available to editors and reviewers. We strongly encourage code deposition in a community repository (e.g. GitHub). See the Nature Research guidelines for submitting code & software for further information.

Data

Policy information about availability of data

All manuscripts must include a data availability statement. This statement should provide the following information, where applicable:

- Accession codes, unique identifiers, or web links for publicly available datasets
- A list of figures that have associated raw data
- A description of any restrictions on data availability

All datasets analysed during the production of TIAToolbox, except for one private oral dysplasia cohort dataset for HoVer-Net+, are publicly available. They can be accessed for research and non-commercial use at the following web addresses:
• The Cancer Genome Atlas (TCGA): https://www.cancer.gov/tcga
• PanNuke: https://warwick.ac.uk/fac/cross_fac/tia/data/pannuke
• PatchCamelyon (PCam): https://github.com/basveeling/pcam
• Kather 100k: https://zenodo.org/record/1214456
• Kumar (MoNuSeg Subset): https://monuseg.grand-challenge.org/
• MoNuSAC: https://monusac-2020.grand-challenge.org/

• CoNSeP: https://warwick.ac.uk/fac/cross_fac/tia/data/hovernet/
The private oral dysplasia cohort dataset is not available because we do not currently have ethical approval to share this dataset but the trained model is already published.

Field-specific reporting

Please select the one below that is the best fit for your research. If you are not sure, read the appropriate sections before making your selection.

☒ Life sciences ☐ Behavioural & social sciences ☐ Ecological, evolutionary & environmental sciences

For a reference copy of the document with all sections, see nature.com/documents/nr-reporting-summary-flat.pdf

Life sciences study design

All studies must disclose on these points even when the disclosure is negative.

Sample size	Publicly available or previously published datasets were used. Hence the sample size was dependent on the data availability.
Data exclusions	Publicly available or previously published datasets were used.
Replication	Models were trained on multiple datasets. Previously published results were also replicated.
Randomization	Original randomized splits were used for publicly available datasets were used where possible. When performing training with the Kather 100k dataset, we used an 80/20 split on the non-normalized data variant due to the availability of only stain-normalized test data. We used non-normalized data to improve generality and computational efficiency of the algorithm. If original data splits were not available, a randomized split was used.
Blinding	Publicly available or previously published datasets were used.

Reporting for specific materials, systems and methods

We require information from authors about some types of materials, experimental systems and methods used in many studies. Here, indicate whether each material, system or method listed is relevant to your study. If you are not sure if a list item applies to your research, read the appropriate section before selecting a response.

Materials & experimental systems

n/a	Involved in the study	
☒	☐	Antibodies
☒	☐	Eukaryotic cell lines
☒	☐	Palaeontology and archaeology
☒	☐	Animals and other organisms
☐	☒	Human research participants
☐	☒	Clinical data
☒	☐	Dual use research of concern

Methods

n/a	Involved in the study	
☒	☐	ChIP-seq
☒	☐	Flow cytometry
☒	☐	MRI-based neuroimaging

Human research participants

Policy information about studies involving human research participants

Population characteristics	Publicly available or previously published datasets were used.
Recruitment	Publicly available or previously published datasets were used. No recruitment was required.
Ethics oversight	N/A. Publicly available or previously published datasets were used.

Note that full information on the approval of the study protocol must also be provided in the manuscript.

Clinical data

Policy information about clinical studies

All manuscripts should comply with the ICMJE guidelines for publication of clinical research and a completed CONSORT checklist must be included with all submissions.

Clinical trial registration	N/A

Study protocol	N/A
Data collection	N/A
Outcomes	N/A
